# Exploring the Application of AI and Extended Reality Technologies in Metaverse-Driven Mental Health Solutions: Scoping Review

**DOI:** 10.2196/72400

**Published:** 2025-08-19

**Authors:** Aliya Tabassum, Ibrahim Ghaznavi, Alaa Abd-Alrazaq, Junaid Qadir

**Affiliations:** 1 Computer Science and Engineering Department College of Engineering Qatar University Doha Qatar; 2 Information Technology University Lahore Pakistan; 3 AI Center for Precision Health Weill Cornell Medical College in Qatar Doha Qatar

**Keywords:** augmented reality, AR, virtual reality, VR, psychotherapy, extended reality ethics, XR ethics, digital therapeutics, cognitive behavioral therapy, immersive technology, telehealth, mental wellness, virtual interventions

## Abstract

**Background:**

Mental health systems worldwide face unprecedented strain due to rising psychological distress, limited access to care, and an insufficient number of trained professionals. Even in high-income countries, the ratio of patients to health care providers remains inadequate to address demand. Emerging technologies such as artificial intelligence (AI) and extended reality (XR) are being explored to improve access, engagement, and scalability of mental health interventions. When integrated into immersive metaverse environments, these technologies offer the potential to deliver personalized and emotionally responsive mental health care.

**Objective:**

This scoping review explores the state-of-the-art applications of AI and XR technologies in metaverse frameworks for mental health. It identifies technological capabilities, therapeutic benefits, and ethical limitations, focusing on governance gaps related to data privacy, patient-clinician dynamics, algorithmic bias, digital inequality, and psychological dependency.

**Methods:**

A systematic search was conducted across 5 electronic databases—PubMed, Scopus, IEEE Xplore, PsycINFO, and Google Scholar—for peer-reviewed literature published between January 2014 and October 2024. Search terms included combinations of “AI,” “XR,” “VR,” “mental health,” “psychotherapy,” and “metaverse.” Studies were eligible if they (1) involved mental health interventions; (2) used AI or XR within immersive or metaverse-like environments; and (3) were empirical, peer-reviewed articles in English. Editorials, conference summaries, and articles lacking clinical or technical depth were excluded. Two reviewers independently screened titles, abstracts, and full texts using predefined inclusion and exclusion criteria, with Cohen κ values of 0.85 and 0.80 indicating strong interrater agreement. Risk of bias was not assessed due to the scoping nature of the review. Data synthesis followed a narrative approach.

**Results:**

Of 1288 articles identified, 48 studies met the inclusion criteria. The included studies varied in design and scope, with most studies conducted in high-income countries. AI applications included emotion detection, conversational agents, and clinical decision-support systems. XR interventions ranged from virtual reality–based cognitive behavioral therapy and exposure therapy to avatar-guided mindfulness. Several studies reported improvements in patient engagement, symptom reduction, and treatment adherence. However, many studies were limited by small sample sizes, single-institution settings, and lack of longitudinal validation. Ethical risks identified included opaque algorithmic processes, risks of psychological overdependence, weak data governance, and the exclusion of digitally marginalized populations.

**Conclusions:**

AI and XR technologies integrated within metaverse settings represent promising tools for enhancing mental health care delivery through personalization, scalability, and immersive engagement. However, the current evidence base is limited by methodological inconsistencies and a lack of long-term validation. Future research should use disorder-specific frameworks; adopt standardized efficacy measures; and ensure inclusive, ethical, and transparent development practices. Strong interdisciplinary governance models are essential to support the responsible and equitable integration of AI-driven XR technologies into mental health care. The narrative synthesis limits generalizability, and the absence of a risk of bias assessment hinders critical appraisal.

## Introduction

### Overview of Artificial Intelligence and Extended Reality in Mental Health

The convergence of artificial intelligence (AI) and extended reality (XR) technologies is revolutionizing mental health care, shifting it from static, clinician-driven interventions to dynamic, immersive therapeutic experiences. AI-driven XR (AI-XR) technologies hold transformative potential in addressing the global mental health crisis, particularly in underserved and remote populations. AI-powered chatbots, digital triage systems, and virtual agents offer scalable, 24/7 support without the need for in-person consultations [1-3]. Simultaneously, XR platforms—particularly in the context of the metaverse—create digital therapeutic environments that enable real-time remote interactions between therapists and patients. These networked ecosystems bypass geographic and socioeconomic barriers, enabling broader access to care and peer support [4]. Notably, metaverse-based clinics and virtual reality (VR) teletherapy rooms are now being explored as accessible alternatives to traditional clinical settings [5,6].

XR serves as a gateway to the metaverse, encompassing immersive technologies such as VR, augmented reality (AR), and mixed reality (MR). Each of these modalities offers distinct capabilities. AR overlays digital content, such as images and sounds, onto the physical world through devices such as smartphones or AR glasses, enhancing real-world experiences [7]. Similarly, MR blends the physical and digital environments, allowing real and virtual elements to interact in real time, fostering deep engagement [8]. However, VR differs from AR and MR in that it fully immerses users in a computer-generated environment, completely replacing the physical world.

Among XR technologies, VR has emerged as the most widely used for digital psychotherapeutic purposes, facilitating treatments such as exposure therapy, mindfulness training, and cognitive behavioral interventions. Using VR headsets equipped with motion-tracking sensors and controllers, users can immerse themselves in richly designed 3D therapeutic environments where they interact with digital objects and navigate scenarios through embodied experiences. These virtual spaces are meticulously crafted to deliver multisensory interventions—combining 360° visuals, ambient soundscapes, haptic feedback, and even olfactory cues—enabling avatar embodiment, teleportation, and emotional immersion that fosters deep cognitive and affective engagement [1,9].

AI plays a crucial complementary role in this ecosystem, powering tools such as therapy chatbots, emotion recognition algorithms, and decision-support systems [1-3,10,11]. The most notable advancements have been in large language model (LLM)–powered conversational agents, which provide scalable, interactive, and accessible mental health support. Recent breakthroughs, including GPT-based models, Llama, and others, have significantly enhanced AI-driven mental health interventions, making them more responsive and contextually aware [12,13]. By integrating AI-driven applications within XR environments, mental health interventions can become more personalized, adaptive, and accessible than ever before.

The promise of AI-XR in mental health lies not merely in its technical capabilities but also in its ability to democratize access to care. By transcending the constraints of traditional in-person therapies, these technologies open pathways for addressing mental health disparities, particularly in underserved regions [14-16]. They provide patients with tailored interventions that adapt to individual needs, creating an unprecedented synergy between technological precision and therapeutic intent [16-19]. Studies highlight the value of AI-XR technologies in enhancing mental health treatments by creating adaptive, immersive environments that foster emotional regulation and support cognitive therapies [7]. In these environments, patients interact not only with AI agents and therapists but also with avatars of peers, family, or caregivers. This social dimension adds a unique layer to therapy, enabling collaborative care and community engagement across cultural and geographic divides [4,20]. Frameworks such as schema therapy have demonstrated how virtual settings promote deeper emotional and cognitive engagement, helping users confront and adapt to challenging scenarios [21]. These technologies have also been shown to hold promise in addressing psychiatric care through metaverse platforms, which facilitate personalized psychiatric tools and virtual medical training, albeit with challenges related to accessibility and socioeconomic disparities [7,22].

### Emerging Opportunities and Challenges

MR environments have been explored for their capacity to create adaptable therapeutic contexts that encourage social interactions and emotional regulation, potentially addressing gaps in conventional therapies [8]. In their research, photorealistic virtual avatars were used by Ghaznavi et al [23] to enhance the efficacy of self-attachment psychotherapy in treating patients with symptoms of chronic anxiety and depression [24]. Another study, by Wei et al [25], investigated the effects of virtual avatar’s facial expressions and animations on paranoid patients. In addition, Freeman et al [9] used an automated immersive VR platform to treat patients with symptoms of persecutory delusions. Further advancements in telerehabilitation using AI-XR technologies are also underway, with studies suggesting that they may soon rival or surpass traditional rehabilitation methods. However, the need for long-term validation remains pressing [26].

The integration of AI-XR technologies in the metaverse has also opened up new possibilities for treating mental health conditions, including autism and posttraumatic stress disorder (PTSD) [5,6]. These technologies offer promising new approaches but remain under exploration regarding their long-term effects, such as the risk of dependency, cognitive impacts, and sustainability within immersive therapeutic environments [20]. By incorporating multiple sensory stimuli—such as sight, touch, sound, and smell—into therapeutic VR and AR settings, these applications engage various brain regions, which in turn can improve emotional regulation, cognitive processing, and even neural reprogramming. Neuroplasticity is leveraged to help patients rewire their neural circuits, fostering healthier reactions to trauma and stress [27].

These technological advances not only enhance user experiences but also signal the advent of Web3, where the human-machine interface is envisioned as a seamless integration of physical, digital, and spatial dimensions. The metaverse thus holds a dual significance for mental health. On the one hand, it presents unparalleled opportunities to expand therapeutic access and foster collaborative care across cultural and geographic boundaries. On the other hand, it poses risks, including potential harm to vulnerable populations and the erosion of critical human elements in mental health care [20]. Despite the growing body of research on AI-XR’s applications, there remains a conspicuous gap in actionable regulatory frameworks that address these challenges. Equally underexplored are the long-term societal impacts of integrating immersive technologies into mental health care, including their influence on patient-therapist dynamics and the broader ethics of care delivery. Addressing these gaps requires a multidisciplinary lens that unites technological innovation with philosophical rigor.

To ensure consistency throughout this review, we defined some key terms (Textbox 1 [7,8,14,28-30]).

**Key terminology.**

- AI-driven: refers to applications leveraging artificial intelligence (AI) techniques—including machine learning, natural language processing, and computer vision—to enhance mental health interventions. These include AI-powered diagnostic tools, chatbots, and personalized treatment recommendations [14].
- Virtual reality (VR): a fully immersive digital environment that replaces the user’s real-world surroundings. VR is typically experienced through head-mounted displays and is used in mental health for exposure therapy, relaxation training, and simulations of social scenarios [30].
- Augmented reality (AR): an interactive experience where digital elements are overlaid onto the real-world environment, usually via mobile devices or AR glasses [31]. AR in mental health can support therapeutic activities by enhancing real-world objects with visual or auditory cues.
- Mixed reality (MR): a hybrid form that merges the physical and digital worlds, allowing real-time interaction between real and virtual elements. MR is increasingly used in therapy to enable more naturalistic interactions in digitally enhanced environments [7,8].
- Extended reality (XR) enabled: encompasses applications using XR technologies, which include VR, AR, and MR [8]. XR-enabled interventions offer immersive therapeutic experiences, exposure therapy, and skill-building exercises in interactive environments.
- Metaverse based: describes mental health interventions conducted within immersive, networked virtual spaces that enable real-time interactions [28]. These platforms integrate AI and XR to facilitate remote therapy, peer support, and social engagement, creating digital therapeutic ecosystems.
- AI-driven XR (AI-XR): a term used to describe the integration of AI and XR technologies for mental health applications. AI-XR solutions combine AI-driven personalization, real-time data analysis, and adaptive interventions with immersive XR environments to create intelligent, interactive, and responsive therapeutic experiences [29,30].

### Research Questions

#### Overview

This paper presents a scoping review combined with a state-of-the-art analysis of AI-XR technologies in mental health. It examines their benefits, challenges—across ethical, legal, technological, and societal dimensions—and potential future directions. By identifying research gaps, it provides actionable recommendations for the ethical integration of AI-XR and metaverse technologies into psychotherapeutic practices, aiming to support researchers, policy makers, and practitioners in maximizing their potential while minimizing risks.

#### Hypothesis

AI-XR technologies have the potential to enhance therapeutic outcomes, increase accessibility, and transform mental health treatment through immersive, AI-driven interventions. However, their adoption poses substantial ethical, societal, and regulatory challenges that must be addressed to ensure their responsible and equitable integration into psychotherapy.

This scoping review addresses the following research questions (RQs):

1. What are the key applications and associated benefits of AI-XR technologies in mental health and psychotherapy? This question seeks to explore how AI-XR technologies are being used across various therapeutic domains, including exposure therapy for phobias; cognitive behavioral therapy (CBT) for depression, anxiety, PTSD, and obsessive-compulsive disorder (OCD); as well as pain management, schizophrenia treatment, social skills training (eg, autism and social anxiety), mindfulness techniques, trauma recovery, neurofeedback, and cognitive rehabilitation. It also examines how these applications leverage the unique capabilities of AI-XR to improve therapeutic outcomes and address diverse mental health challenges.
2. What are the ethical and societal challenges and barriers to adopting AI-XR technologies in mental health therapy? These challenges include the digital divide, cybersickness, data privacy and security concerns, informed consent issues, potential effects on the therapeutic relationship, and long-term psychological effects on mental health outcomes.
3. What regulatory frameworks are necessary to ensure ethical and equitable integration of AI-XR technologies in mental health treatment? This includes addressing challenges such as equitable access, data privacy, and algorithmic bias, while safeguarding the humanistic elements essential to psychotherapy. Emphasis is placed on integrating comprehensive governance models, clinical oversight, and ethical principles to maintain care integrity and uphold human dignity.

The selection of RQs was guided by a systematic review of existing literature and an analysis of unresolved challenges in AI-XR applications for mental health. While prior studies have explored specific use cases (eg, VR-based exposure therapy or AI-driven diagnostics), a comprehensive synthesis of AI-XR’s broader therapeutic potential, ethical challenges, and governance needs remains lacking.

- RQ1 (applications and benefits) was chosen to consolidate fragmented evidence on the role of AI-XR across multiple therapeutic domains, as existing studies often focus on isolated interventions without considering their cross-cutting impact on different disorders.
- RQ2 (ethical and societal challenges) addresses critical concerns such as privacy risks, data biases, and patient-therapist dynamics, which are frequently mentioned in studies but lack a systematic assessment of their implications for mental health treatment.
- RQ3 (regulatory frameworks) was formulated in response to gaps in governance strategies, as current regulatory discussions on AI ethics often overlook the immersive and deeply personal nature of mental health interventions in XR environments.

By answering these questions, this study aimed to bridge theoretical insights with practical considerations, offering actionable recommendations for researchers, clinicians, and policy makers.

The structure of the paper is as follows: the Methods section details the scoping review methodology, including study selection criteria, search strategies, data extraction, and synthesis methods. The Results section categorizes the findings based on the benefits, ethical dilemmas, and societal impacts of AI-XR technologies. In the Discussion section, the results are analyzed in relation to the RQs, emphasizing research gaps in areas such as long-term mental-health outcomes, equity, and governance frameworks. The **Limitations section** discusses key constraints of the study and identifies critical areas for future research. Finally, the **Conclusions section** summarizes the main insights and offers a call to action for stakeholders to ensure the ethical integration of AI-XR technologies in mental health contexts.

## Methods

### Overview

To conduct this scoping review, we followed the guidelines outlined in PRISMA-ScR (Preferred Reporting Items for Systematic Reviews and Meta-Analyses extension for Scoping Reviews) [32]. The PRISMA-ScR checklist relevant to this review is provided in Multimedia Appendix 1 [32]. The subsequent subsections offer a detailed explanation of the methods used in this review.

### Search Strategy

The search was conducted across 5 academic databases to identify relevant literature on integrating AI-XR and metaverse technologies in mental health treatments. These databases included PubMed, PsycINFO, IEEE Xplore, Scopus, and Google Scholar. The search process began on September 16, 2024, using a variety of keywords, and was iteratively refined based on the retrieval of studies. This search was concluded on November 25, 2024. Our search criteria comprised 3 main categories of terms—metaverse-related terms (VR, AR, XR, and immersive technologies); psychotherapy-related terms (anxiety, depression, stress, cognitive, posttraumatic stress, schizophrenia, eating disorder, phobia, and autism); and outcome-related terms (benefit, advantage, effective, impact, improve, efficacy, challenge, barrier, privacy, safety, and regulate). The detailed search queries used for each database are provided in Multimedia Appendix 2.

### Study Eligibility Criteria

This review focused on the studies that investigated the application of metaverse and XR technologies in mental health treatment, with particular emphasis on therapeutic applications such as psychotherapy, CBT, or treatments for psychological disorders. Articles using AI (including machine learning and deep learning) and XR technologies (VR, AR, MR, or metaverse-based systems) in mental health treatment. However, we have excluded articles that solely focus on AI technologies, ensuring that our review includes studies that explore the intersection of AI with VR, XR, AR, or the metaverse. The inclusion and exclusion criteria are shown in Textbox 2.

**Inclusion and exclusion criteria for selecting eligible articles.**

**Inclusion criteria**

- Research articles: peer-reviewed empirical and review articles only
- Publication date: published after 2014 to ensure relevance to modern artificial intelligence (AI) and extended reality (XR) advancements
- Mental health context: articles addressing AI or XR interventions for mental health treatment, psychotherapy, or digital therapeutics
- Ethical issues: studies discussing ethical and regulatory implications of AI or XR in mental health care

**Exclusion criteria**

- Nonresearch articles: editorials, commentaries, policy papers, abstracts, or conference summaries without empirical findings
- Irrelevant focus: studies primarily focused on AI or XR without a clear application to mental health or the metaverse
- Low-quality sources: preprints, non–peer-reviewed literature, or articles with insufficient methodological transparency

In terms of predicted outcomes, our focus was centered on 3 key categories: (1) articles that discussed the benefits and applications of the metaverse, VR, XR, and AR in mental health treatments, highlighting their potential to enhance therapeutic outcomes; (2) studies addressing the risks, social problems, and broader implications arising from the intersection of these technologies with mental health practices; and (3) research that emphasized the importance of developing regulatory frameworks, strategies, and future guidelines to ensure these immersive technologies are used safely and effectively, minimizing negative implications while maximizing their benefits. However, studies focused solely on technical aspects of AI or XR without direct application to mental health treatment were excluded. Similarly, we eliminated the articles that addressed applications of AI-XR technologies not related to mental health, such as general health care, education, or gaming.

This study adopts a scoping review methodology to systematically explore the intersection of immersive technologies—such as the metaverse, VR, XR, and AR—with mental health applications. To ensure comprehensiveness, we included peer-reviewed articles, theses, dissertations, experimental studies, systematic reviews, and conference papers published in English, with constraints on publication year from 2014 to 2024. Non–peer-reviewed articles, preprints, reviews, opinion pieces, editorials, case studies, conference abstracts, and protocols were excluded. This approach allows for a structured synthesis of current evidence and insights, emphasizing themes and gaps critical to advancing this interdisciplinary field.

### Study Process

The study selection process was carried out in 3 phases. First, duplicates were removed from the retrieved studies using a simple Microsoft Excel duplicate removal tool. The titles and abstracts of the remaining articles were then screened for relevance. In the final phase, the full texts of the shortlisted studies were thoroughly evaluated. The selection process was conducted independently by 2 reviewers. Disagreements during the second and third phases were resolved through discussion. Cohen κ was calculated to assess interrater agreement, yielding a score of 0.85 for the “title and abstract” screening phase and 0.80 for the full-text reading phase. Studies were excluded if they did not specifically focus on AI and XR in mental health or psychotherapy. Priority was given to studies that provided a clear discussion of benefits, outcomes, ethical considerations related to patient data, privacy, and therapeutic use of immersive technologies. Articles that did not adequately address benefits, outcomes, and ethical or privacy concerns were excluded.

### Data Extraction

The data extraction process involved 2 independent reviewers using a detailed form in Microsoft Excel to gather key information from the studies. Extracted data included study characteristics such as author, publication year, type, and methodology, alongside details of settings (eg, virtual environments, clinics, or online platforms) and findings related to the applications and impacts of AI, XR, VR, AR, and metaverse technologies in mental health. Additional data encompassed the technologies and tools used (eg, AI-powered chatbots and virtual therapy platforms), AI methods such as machine learning and natural language processing, and the studies’ focus on psychotherapy and broader mental health treatments, including conditions such as anxiety, depression, and PTSD. The extracted data form is shown in Multimedia Appendix 3. Discrepancies between the reviewers were resolved through discussion.

### Data Synthesis

The data synthesis process used a narrative approach to analyze and present the findings from the included studies. Key aspects, such as study metadata (eg, publication year and country), were outlined, and the distribution of studies across RQs was calculated and visually represented using figures and tables. The synthesis delved into the applications of AI and XR technologies in mental health, highlighting their roles in enhancing psychotherapy approaches, including AI-driven CBT and the use of AI-powered psychotherapy chatbots. Ethical and social implications, such as privacy concerns, bias, fairness, and cybersickness, were also explored. Furthermore, regulatory frameworks were reviewed, emphasizing transparency, data protection, and security in the use of AI-XR technologies for mental health. The synthesis provided insights into both the benefits and challenges of these technologies, incorporating data on clinical outcomes, patient engagement, therapist feedback, and evaluation metrics, while proposing strategies and guidelines for their effective and ethical integration into mental health care practices.

## Results

### Search Results

**Figure 1:**
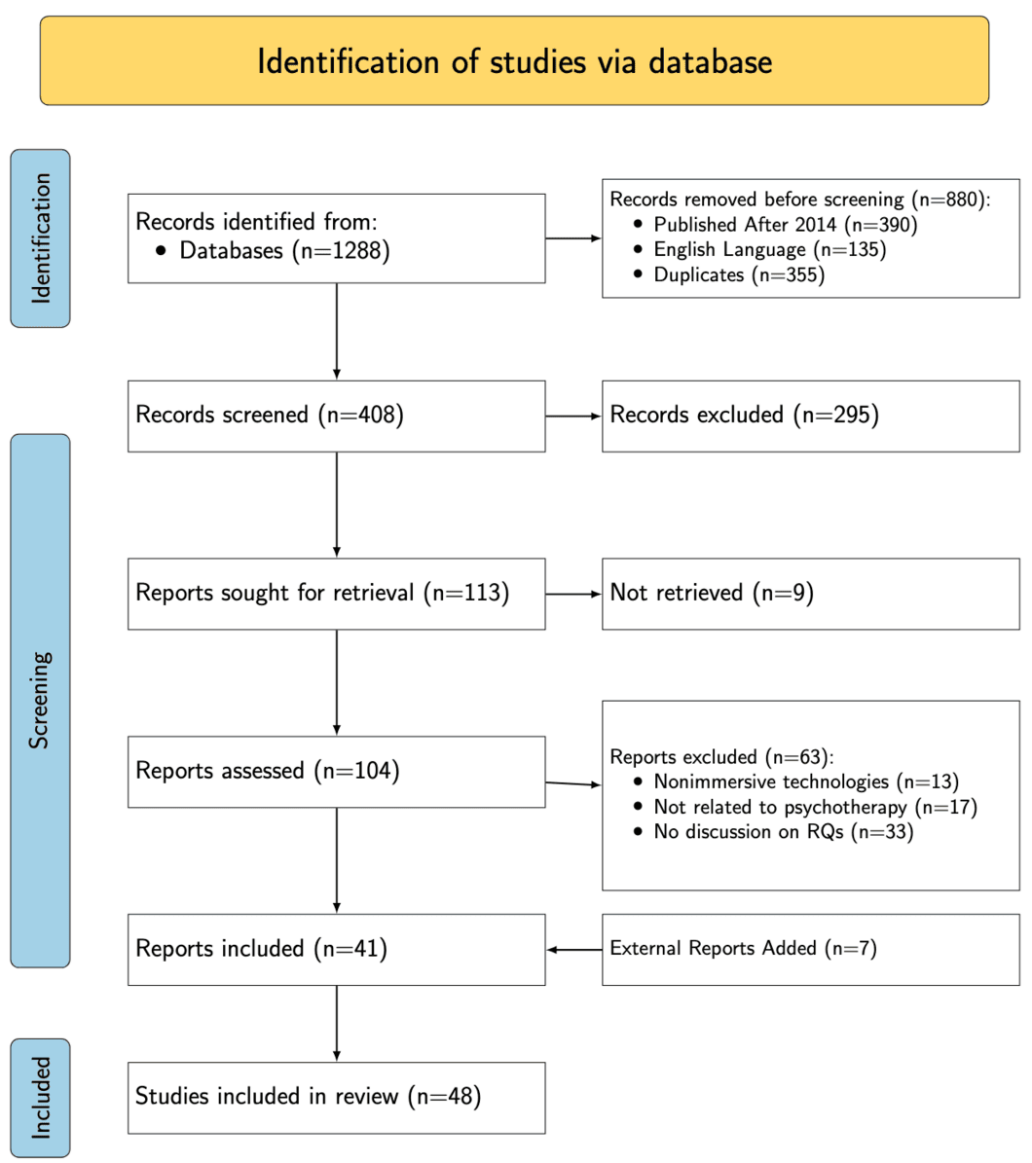
PRISMA-ScR chart illustrating the article selection process. RQ: research question.

Figure 1 presents the search process and results from the preselected databases, yielding a total of 1288 records. Articles published before 2014 were excluded, resulting in 898 records. Of these, we refined articles for only English language, which resulted in elimination of 135 articles. Then, from these, 355 duplicates were identified and removed using Microsoft Excel. Screening the titles and abstracts of the remaining 408 articles led to the exclusion of 295. Out of the 113 articles remaining, 9 full texts were unavailable. Following a detailed full-text review of the accessible articles, 41 were excluded for various reasons outlined in Figure 1. In addition, 7 articles were identified through backward and forward referencing. In total, 48 articles were included in this review as shown in Table 1.

**Table 1:** Research question 3: integrated strategies, regulatory frameworks, and ethical issues for artificial intelligence–driven extended reality (AI-XR) technologies in mental health and psychotherapy.

Strategy or issue	Implications and regulatory frameworks	Mitigation strategy and references
Expand telerehabilitation through AI-XR	Long-term regulatory monitoring and efficacy assessments; risk of data privacy breaches, misuse, or cyberattacks	Security measures, confidentiality protocols, and data anonymization [26,33]
Incorporate blockchain for secure health care	Regulatory frameworks for data privacy and secure transactions; address digital inequality and ensure equitable access	Subsidies for disadvantaged groups, diverse AI training, and transparent regulations [15,34]
Develop embodied AI as complementary therapy	Ethical frameworks for AI risk assessments; bias in AI algorithms leading to discrimination	Diverse, unbiased, and transparent training of AI models [14,35]
Address accessibility in metaverse psychiatric care	Regulations ensuring equitable access; psychological impact, such as anxiety and overreliance	Clear consent forms, patient education, and regular psychological assessments [7,36]
Revolutionize medical education with metaverse tech	Inclusivity, data privacy, and security regulations; inclusivity issues related to cultural sensitivity	Developing culturally sensitive content, inclusive practices, and clear patient communication [17,37]
Enhance health care with AI-powered medical technology	International regulations on AI and blockchain in health care; regulatory gaps in unverified wellness content	Accountability measures, oversight, and validated platforms for ethical compliance [36,38]
Health care (secure data)	XR, blockchain, DLT: unregulated use, lack of policies	Oversight and validated platforms for secure health care data [15,34]
Leverage digital twins in surgical precision	Regulatory collaboration for safe and equitable use of digital twins; risk of digital inequality	Equitable access through subsidies and transparent data use [39]
Regulate XR-enabled group therapy	Legal protections for privacy, control, and equitable access; risk of bias and exclusion in virtual therapy	Developing inclusive practices and unbiased, transparent regulations [8]

### Characteristics of Included Studies

Table 2 provides an in-depth summary of the 48 articles reviewed in this study, focusing on the applications of AI and XR technologies in mental health treatment. The table categorizes the studies by type—empirical, conceptual, or systematic review—and highlights their main contributions and key findings. Each article’s relevance to the RQs is mapped in the final column, showing how the study contributes to understanding the benefits (RQ1), risks (RQ2), or regulatory frameworks and strategies (RQ3) associated with these technologies.

**Table 2:** Summary of studies in our review: focusing on artificial intelligence–driven extended reality (AI-XR) technologies and their applications in mental health treatment.

Study	Key findings	Relevance
Akhtar [18], 2024	AI-XR for personalized mental health; shortened therapy cycles with VR^a^	RQ^b^1: tailored treatment
Al Dweik et al [40], 2024	Technological challenges and need of human oversight in digital mental health interventions, platforms, and modalities	RQ1 and RQ3: improved clinical outcomes and need for regulatory frameworks
Ali et al [34], 2023	Metaverse system for physician-patient interactions and blockchain for secure data	RQ1: secure health care in metaverse
Arnfred et al [2], 2022	CBT^c^ via group therapy versus VR as potential alternative to in vivo CBT	RQ1: iterative design of VR scenarios with feedback
Benrimoh et al [41], 2022	Impact of metaverse on mental health and teletherapy risks	RQ2: VR risks in mental health
Bhugaonkar et al [39], 2022	VR dependency and privacy risks in therapy	RQ2: long-term mental health outcomes
Blackmore et al [42], 2024	Stress and cognitive load due to use of biosignals in health care and education	RQ1: tracks patient states
Cerasa et al [43], 2024	Metaverse for psychiatry and disorder-specific treatments	RQ1: disorder treatment in metaverse
Chengoden et al [44], 2023	Metaverse adoption in health care with future solutions	RQ2 and RQ3: accessibility barriers
Cho et al [28], 2024	Higher satisfaction metaverse versus in-person counseling	RQ1 and RQ3: therapeutic relationship benefits
Coghlan et al [45], 2023	Potentials and pitfalls of chatbots in health care: issues and recommendations	RQ1 and RQ2: benefits and ethical concerns of chatbots
Costanzo [31], 2024	Conscious use of therapy, psychology, and technology to avoid the risks of addiction or emotional desensitization	RQ3: guidelines to implement AR and VR in therapy
Distor et al [20], 2023	Balances benefits and risks in immersive environments in therapy	RQ1 and RQ3: risks and benefits in therapy
Usmani et al [46], 2022	Future visions and applications of the metaverse in health delivery	RQ1 and RQ2: potential of metaverse for health care and limitations
Fajnerova et al [47], 2024	Application of multiuser virtual environments and VR in mental health versus in-person interventions	RQ1 and RQ2: challenges and benefits associated with VR, including technical demands, usability barriers, and cybersickness
Ferrario et al [48], 2024	Ethical, technical, clinical challenges and recommendations for responsible design and deployment	RQ2: risks of integrating LLMs^d^
Fiske et al [14], 2019	Ethical insights on AI in mental health and decision-making	RQ2 and RQ3: ethical challenges and guidelines
Ford et al [7], 2023	Metaverse applications, accessibility, and ethical risks in psychiatric care	RQ1 and RQ2: therapeutic benefits and challenges
Freeman et al [49], 2019	Automated VR cognitive therapy using an avatar coach for psychological therapy	RQ1: patients find VR environments less intimidating
Guest et al [50], 2023	Identified substantial digital solutions for diagnostics, monitoring, and treatment	RQ1 and RQ2: benefits and limitations
Jiang et al [51], 2024	Automated therapist quality assessment and client adherence prediction based on adherence to CBT protocols	RQ1: role of AI in CBT
Ullah et al [52], 2023	Design for virtual psychotherapy and avatar realism	RQ1, RQ2, and RQ3: design and therapy benefits, challenges, and guidelines
Saredakis et al [53], 2021	AI-XR in telerehabilitation outperforms traditional methods	RQ1: enhances therapeutic outcomes
Meinlschmidt et al [54], 2023	Framework identifying 4 mental health conditions related to the metaverse	RQ1 and RQ2: benefits and risks of immersive treatments
Mitsea et al [55], 2023	Effectiveness of XR digital technologies in health care: self-regulation, mental well-being, and productivity	RQ1: AI-XR technologies role in enhancing mental well-being
Moldoveanu et al [56], 2023	PhoVR Phobia therapy through VR for treating acrophobia, claustrophobia, and public speaking anxiety	RQ1: VR therapy for treating acrophobia, claustrophobia, and public speaking anxiety
Moodley et al [15], 2023	XR and blockchain for secure health data and privacy in remote counseling	RQ1 and RQ2: secures patient data and risks
Nadarasa [17], 2024	Framework for metaverse in health care to preserves human dignity	RQ2: patient-centered ethics
Namkoong et al [35], 2024	Nonverbal immediacy in AI counseling to enhance rapport in virtual settings	RQ3: user experience in AI counseling
Navas-Medrano et al [8], 2023	XR-enabled therapeutic environments to enhances social interaction	RQ1: social benefits in therapy
Obremski and Wienrich [57], 2024	Autonomous VR exposure therapy to improve the psychotherapeutic supply	RQ1 and RQ3: benefits and ethical principles measurable for developers
Olukayode et al [36], 2024	Transformative advancements of mental health, AI therapy, and the need of robust regulations	RQ2 and RQ3: risks and needed regulatory frameworks
Jane Patel [58], 2024	Ethics, privacy, and consent in VR for mental health	RQ2 and RQ3: ethical considerations and guidelines
Pavlopoulos et al [59], 2024	Potential of LLMs in managing anxiety and depression, but ethical issues remain	RQ1 and RQ2: effectiveness, accessibility, and risks of AI tools in mental health care
Radanliev [60], 2024	XR framework for DMT^e^ diversifies mental health therapies	RQ1: XR in alternative therapies
Radanliev [61], 2024	Potential of integrating DMT within XR environments	RQ1: convergence of DMT, XR, and AI in mental health treatment
Riches et al [27], 2024	VR for stress reduction and wellness intervention access	RQ1: stress and wellness benefits
Saudagar et al [37], 2024	AR and VR privacy and access issues in medical education	RQ2: access and equity issues
Schoenberg [62], 2023	Digital disparities and schizophrenia issues in mental health	RQ2: challenges
Sestino and D’Angelo [63], 2023	Avatar use for empathy and trust in health care	RQ1 and RQ2: benefits and challenges
Shao et al [11], 2023	Risks of unregulated wellness content and privacy	RQ2: challenges
Soares et al [64], 2020	Potential psychotherapy using AI and emerging technologies	RQ1: benefits and trends in psychotherapy
Taçgın [10], 2023	A compact virtual therapy environment: IVR prototype called RelaXRoom	RQ1: application of VR therapy
Wang et al [38], 2022	AI-powered medical technology revolutionizes health care infrastructure	RQ1: technological advancement
Xu and Zhuang [65], 2022	Guided and standardized chatbots must be used in psychotherapy	RQ1, RQ2, and RQ3: benefits, risks, and guidelines
Yin et al [21], 2022	Framework for metaverse in schema therapy to support behavioral change	RQ1: immersive therapy benefits
Zafar [66], 2023	Metaverse’s potential to revolutionize the health care	RQ1: use cases of metaverse in health care and therapy
Zhang and Wang [33], 2024	The long-term efficacy of AI-based therapies is questionable	RQ1 and RQ2: potential advantages and challenges

^a^VR: virtual reality.

^b^RQ: research question.

^c^CBT: cognitive behavioral therapy.

^d^LLM: large language model.

^e^DMT: dance movement therapy.

Table 3 provides a comprehensive overview of the distribution of articles across various dimensions, focusing on the year of publication, country of publication, and the RQs addressed in the field of AI-XR applications in mental health, although the search period was from 2014 to 2024. In terms of publication year, 2024 accounts for 48% (23/48) of the studies, signaling a recent surge in AI-XR research in mental health. This is followed by 2023 with 33% (16/48), indicating continued growth. Publications from 2022 represent 13% (6/48), while 2021, 2020, and 2019 each contribute 2% (1/48), reflecting the field’s recent emergence.

**Table 3:** Characteristics of the included studies (N=48).

Features			Studies, n (%)		References
**Year of publication**					
	2024	23 (48)		[8,10,14,17,18,27,31,33,35-37,40,42,43,47,48,51,57-61,66]	
	2023	16 (33)		[7,10,11,15,20,28,34,44,45,50,52,54-56,62,63]	
	2022	6 (13)		[2,21,38,39,41,46,65]	
	2021	1 (2)		[26]	
	2020	1 (2)		[64]	
	2019	1 (2)		[49]	
**Country of publication**					
	Switzerland	13 (27)		[7,8,21,33,34,50,52,55,56,59-61,65]	
	United States	11 (23)		[15,39,42-45,51,62,64-66]	
	United Kingdom	10 (21)		[2,26-28,35,38,46,49,58,65]	
	Canada	4 (8)		[14,41,47,48]	
	China	2 (4)		[17,57]	
	Italy	2 (4)		[31,63]	
	Others (6 countries)	6 (13)		Hungary [20], India [36], Indonesia [18], Singapore [11], South Korea [37], Turkey [10], Germany [55]	
**Publication type**					
	Conference paper	2 (4)		[52,66]	
	Journal article	46 (96)		[2,7,8,10,11,14,15,17,18,20,21,26-28,31,33-51,54-65]	
**RQ** ^a^ **distribution**					
	RQ1	35 (50)		[2,10,14,15,18,20,21,28,31,33-38,40-48,51,53,55-57,60-62]	
	RQ2	23 (33)		[7,15,20,31,34-36,38,39,41,45,46,48,51-55,58,59,62,64]	
	RQ3	12 (17)		[7,14,27,34,37,40,49,51,58-60,62]	

^a^RQ: research question.

In terms of country of publication, the United States leads with 27% (13/48) of publications, followed by Switzerland (11/48, 23%). The United Kingdom contributes 21% (10/48) of publications, underscoring global interest. Other countries such as Canada, China, and Italy account for smaller yet notable shares, while a collective 12.5% comes from Hungary, India, Indonesia, Singapore, South Korea, Turkey, and Germany, highlighting the international scope.

Regarding RQs, RQ1 (benefits of AI-XR in mental health) dominates with 50%, focusing on the positive impacts of these technologies. RQ2 (challenges and limitations) follows with 32.86%, indicating significant attention to obstacles. RQ3 (ethical considerations and future directions) represents 17.14%, showing a lesser focus but still notable interest in the broader implications of AI-XR technologies.

Furthermore, most of the studies are journal articles (46/48, 96%), with conference papers (2/48, 4%) comprising a smaller proportion, underscoring a predominant focus on journal publications.

Textbox 3 [1-3, 7, 8, 10, 11, 14, 18, 20-22, 26, 28, 31, 33, 34, 36, 37, 39-44, 46-48, 51-55, 57-63, 65-80] details the benefits, challenges, and regulatory guidelines for implementing AI-XR technologies in mental health treatments. This table provides an organized overview of the key insights from the studies, summarizing the positive impacts, potential obstacles, and the current state of regulatory frameworks, contributing to a holistic understanding of the field’s progress and the hurdles that still need to be overcome.

**Insights from the scoping review: key benefits, challenges, and regulatory strategies for artificial intelligence–driven extended reality (AI-XR) applications in mental health.**

**Benefits**

- Therapeutic interventions: exposure therapy [2,41]; cognitive restructuring [7,47]; dance movement therapy [60,61]; psychotic disorders treatment [3,8,43,47]; cognitive rehabilitation [8]; anxiety and depression reduction [10,53]
- Support and recovery; trauma recovery [11,53]; addiction recovery [8,10,55]; emotional regulation [1,67]; shared coping strategies [3,67]; real-time feedback [10,42,51];
- Enhanced personalization: adaptive personalized experiences [8,21]; personalized therapy models [18,51]; real-time progress tracking [39]; AI-powered chatbots [65]; embodied AI-driven cognitive behavioral therapy [2,14]
- Technological advancements: immersive interactive experiences [10]; immersive diagnostics [44]; gamified therapy [26]; accessibility in remote regions [1,10,36,67];
- Scalable interventions [10]

**Challenges**

- Privacy and security: data privacy and data breaches [22,40,48,68]: identity verification, trust, and confidentiality [22,40,48,68]; informed consent challenges [58]
- Ethical and societal concerns: algorithmic bias and lack of transparency [7]; digital divide [37,58,66]; accessibility issues and data inequality [37,58,66]; inclusive design gaps [37,58]
- Psychological risks: emotional overload and overreliance [20,21,31,58]; cognitive strain and emotional dependency [21,31,58]; maladaptive escapism [46,54,69,70]; identity dissociation and dual representations [46,54,69,70]
- Technological limitations: virtual harassment and offensive behaviors [20,31]; trust and regulatory issues [31,63]; cybersickness and screen-mediated connection issues [2,71]; autonomy and patient-therapist relationship disruptions [33,65,66,72,73]; cognitive and epistemic limitations of large language models [7,48,57,59,74]; intuition and empathy loss [7,53,57,63]

**Regulatory guidelines and frameworks**

- Ethical standards: fairness, transparency, and accountability [34,75]; standards for explainable AI [34,52]
- Privacy and consent: data privacy regulations [28,62]; reconsent protocols in long-term AI-XR treatments [57,76]
- Content and accreditation: age-based content filters and disclaimers [77]; standardization of clinician accreditation [78]
- Technological strategies: blockchain and distributed ledger technology [34,79]; long-term impact assessments [33,52]
- Global policies
- Digital health policies from the World Health Organization Global Strategy on Digital Health (2020-2025) [80]

### RQ1: Benefits of AI-XR Applications in Mental Health Treatment

#### Overview

The integration of AI and XR technologies has ushered in transformative advancements in mental health treatment [47,50]. These technologies offer distinct advantages in treatment accessibility, personalization, and immersive experiences, addressing a wide range of mental health conditions. This section explores the applications of AI-XR in mental health therapy, focusing on the key benefits, including increased engagement, customization, and the ability to tackle specific psychological conditions effectively.

#### Therapeutic Applications, Interventions, and Outcomes of AI-XR in Mental Health

AI-driven XR has proven particularly effective in exposure therapy, allowing patients to confront and manage phobias such as arachnophobia, social anxiety, and PTSD within controlled virtual environments [41,56]. Studies comparing AI-driven XR therapy (CBT in virtuo) with traditional CBT in vivo for individuals with social anxiety disorder and agoraphobia have demonstrated its effectiveness. Anxiety symptoms were evaluated using the Liebowitz Social Anxiety Scale and the Mobility Inventory for Agoraphobia, with scores standardized through the percentage of maximum possible method for direct comparison. Posttreatment findings indicate that patients receiving AI-enhanced XR therapy exhibited lower anxiety symptoms than those undergoing conventional therapy, underscoring the potential of AI-integrated XR interventions in mental health treatment [2]. Similarly, in neurosurgical planning, AR navigation techniques have been applied to improve surgical precision, particularly in spine fixation procedures. AR-assisted surgical navigation has led to higher accuracy in screw placement and a reduced incidence of cortical breaches, highlighting its potential to enhance surgical outcomes [18].

These platforms also facilitate cognitive restructuring and deliver personalized, engaging CBT experiences, showing promising outcomes in reducing symptoms of depression, OCD, and trauma-related disorders [7,19,47]. In addition, AI-XR aids in chronic and acute pain management by enhancing relaxation and providing psychological distraction. In addition, it supports the treatment of psychotic disorders (eg, schizophrenia) through virtual environments and interactive avatars, offering safe spaces for individuals with autism and social anxiety to practice social skills [3,8,43,47,62]. Cognitive rehabilitation through AR is also revolutionizing treatments for attention-deficit/hyperactivity disorder, dementia, and brain injuries, while AI-XR tools support addiction recovery and improve mental well-being in older adult populations [8,10,41,55,62].

AI-XR systems are further effective in fostering mindfulness, emotional regulation, and stress management, benefiting patients with anxiety, depression, and general stress through immersive, personalized environments [1,19,28,42,44,67]. For instance, a study compared metaverse-based and in-person counseling in 60 university students, with 32 choosing metaverse-based counseling and 28 choosing in-person counseling. Psychological symptoms were measured before, after, and during follow-up, while therapeutic relationships and satisfaction were assessed during the session and after the session. Both modalities showed similar symptom reduction, with metaverse counseling improving depression, anxiety, and social support deficits. Findings support its feasibility for telemental health, warranting further research on its broader applications [28]. XR combined with brain-computer interfaces enhances emotional self-regulation and relaxation, particularly for patients with depression, anxiety, and stress disorders [27,55]. VR-based interventions are particularly impactful in trauma recovery, providing controlled environments for processing traumatic memories and reducing PTSD symptoms [11].

VR platforms such as multiuser VR or multiuser virtual environment create shared spaces for practicing coping strategies, fostering emotional stability and therapeutic engagement [3,67]. Studies on digital multiuser interventions in mental health care report positive therapeutic effectiveness, particularly in reducing symptoms of depression and anxiety. Primary outcome measures include symptom reduction, engagement, usability, and acceptability. Patient feedback highlights that digital platforms help overcome social barriers and foster a sense of community. While some studies show partial symptom reduction, randomized controlled trials consistently demonstrate that these interventions effectively alleviate symptoms, making them a promising alternative to traditional therapy [47]. Embodied AI systems and immersive metaverse environments further enhance therapeutic outcomes by enabling adaptive, personalized experiences for complex conditions such as personality disorders [8,21]. In addition, AI-enhanced avatars and digital twins improve treatment precision through real-time progress tracking and predictive analytics [39]. Structured VR platforms, such as TRIPP, use immersive visuals and soundscapes to reduce anxiety and depression, highlighting XR’s ability to replicate therapy sessions with high levels of presence and customization [10]. These findings are summarized in Table 4.

**Table 4:** Artificial intelligence (AI)–driven extended reality (XR) interventions and reported outcomes.

AI-XR intervention	Sample size	Outcome measures	Reported outcomes
AI-XR therapy for social anxiety and agoraphobia	302 participants	Liebowitz Social Anxiety Scale and Mobility Inventory for Agoraphobia (converted to percentage of maximum possible)	Symptom reduction and adherence rates [2]
Enhanced exposure therapy using virtual reality	Not specified	Symptom reduction in phobias, posttraumatic stress disorder, obsessive-compulsive disorder, etc	Comparable to traditional exposure therapy but with added benefits (eg, safety and real-time customization) [51]
Augmented reality for neurosurgical planning	Not specified	Accuracy of screw placement, cortical breach rate	Improved precision in spine fixation procedures [18,81]
Metaverse-based versus in-person counseling for psychological symptoms	60 students (32 metaverse and 28 in-person)	Psychological symptoms (before, after, and during follow-up), therapeutic relationships (during the session), and satisfaction (after the session)	Metaverse counseling improved depression, anxiety, and social support deficits [28]
Virtual worlds	Not specified	Symptom reduction (depression and anxiety), engagement, usability, and long-term benefits	Positive overall; symptom reduction in videoconferencing or chat rooms [47]
AI-based metaverse consultation environment	762 participants	User counseling satisfaction and nonverbal immediacy behavior	Enhanced user satisfaction [35]
Medical chatbots (low human-like interaction) versus physicians’ avatars in the metaverse (high human-like interaction)	689 participants	Intention to use digital-based health care services	Higher human-like interaction (physicians’ avatars) increases intention to use via perceived anthropomorphism [63]

#### Immersion, Personalization, and Accessibility of Therapies

AI-powered XR environments enable personalized therapy models tailored to individual needs [18,51]. For example, RelaXRoom integrates meditation, peer-to-peer, and group therapy with mood-based customization, while biosensing technologies (eg, eye-tracking and electrodermal activity monitoring) provide real-time feedback to dynamically adjust therapy [10,42,51]. Innovations such as dance movement therapy in XR settings have shown notable reductions in anxiety and depression symptoms, making mental health treatment more accessible, particularly in remote or underserved regions [60,61]. VR exposure therapy (VRET) has emerged as a powerful alternative to traditional in vivo exposure therapy, addressing logistical and accessibility challenges. VRET provides a safe, customizable, and immersive environment, demonstrating comparable efficacy in reducing symptoms of phobias, PTSD, and OCD while offering added benefits such as real-time customization and increased patient comfort [51,81]. Applications such as **multimodal motion-assisted memory desensitization and reconsolidation** leverage multisensory inputs for trauma-focused therapies, demonstrating VR’s efficacy in reducing psychological distress [1,2,10,36,67].

Similarly, a study examined the impact of AI agents’ nonverbal immediacy behavior on user satisfaction in counseling settings within a metaverse consultation environment. Using 762 valid responses from participants recruited via Amazon Mechanical Turk, the study found that AI agents displaying nonverbal immediacy behavior significantly enhanced user satisfaction [35]. This effect was mediated by positive expectancy violation and rapport, with serial mediation further strengthening the relationship [35].

AI-powered psychotherapy chatbots (eg, Woebot, Wysa, and Joyable) leverage natural language processing to deliver structured, evidence-based interventions such as CBT, addressing barriers such as financial, geographical, and societal constraints [65]. These chatbots provide accessible, confidential, and timely support, with tools such as Woebot aiding in managing depression, anxiety, addiction, and loneliness through pattern recognition in text and emojis [82]. Integrating chatbots within XR environments enhances their therapeutic potential by creating immersive, interactive experiences, positioning them as vital components in extending mental health care into the metaverse [28,83]. However, challenges remain, such as generating real-time, contextually appropriate responses in immersive environments that require multimodal understanding of voice, gestures, and avatar behaviors, which can impact user trust and therapeutic efficacy. A study with 689 participants examined the impact of human-like interaction in digital health care services, comparing medical chatbots (low interaction) to physicians’ avatars in the metaverse (high interaction). Findings revealed that higher human-like interaction increased the intention to use digital health care services through perceived anthropomorphism. Emotional receptivity moderated this effect, with a significant impact only among participants with high emotional receptivity. A moderated-mediation analysis confirmed a positive indirect effect of human-like interactions on intention to use [63].

**Figure 2:**
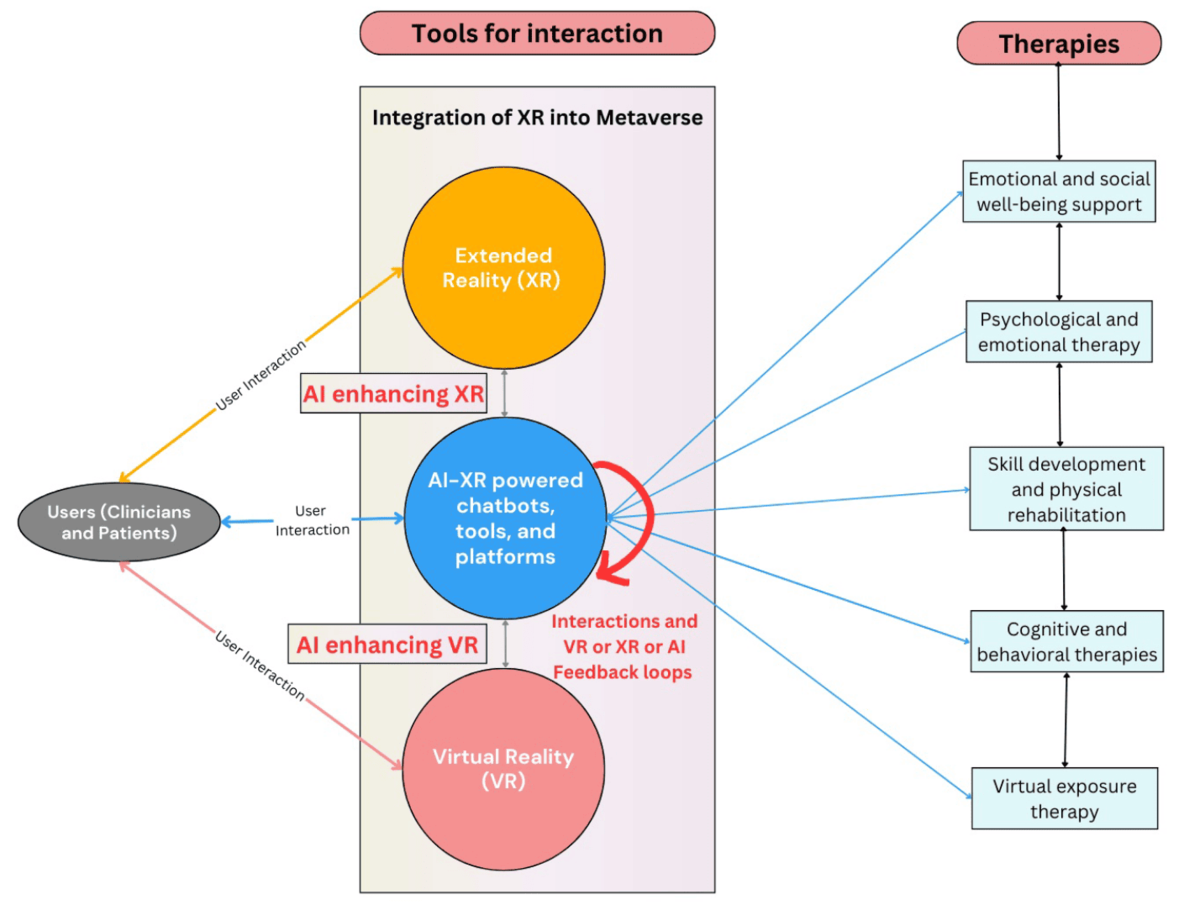
Conceptual diagram showing interactions between users, artificial intelligence (AI), and immersive technologies for mental health therapies. VR: virtual reality; XR: extended reality.

Table 5 summarizes how different AI-XR technologies are being applied in various therapeutic areas. Figure 2 presents a visual representation of the interaction between tools, applications, and users in the context of AI and immersive technologies for mental health interventions. On the left, essential tools such as AI-powered chatbots, VR, and XR are depicted as the foundational technologies driving these advancements. On the right, specific therapeutic applications, including CBT and VRET, are highlighted as outcomes enabled by these tools. Positioned centrally on the left, the “users” node represents the interaction of patients and clinicians with these technologies, connected by arrows to emphasize their active participation. We have listed the benefits that AI-XR applications bring to mental health and psychotherapy treatments in Textbox 2.

**Table 5:** Research question 1: overall artificial intelligence–driven extended reality (AI-XR) applications in mental health treatments.

AI-XR technology	Application	Mental health domain	Reference
VR^a^	VR exposure therapy	Treatment for phobias, PTSD^b^, social anxiety, and trauma recovery	[2,11,41,56]
VR	Multiuser VR (group sessions)	Eating disorders, social anxiety, group therapy, and collaborative coping strategies	[8,43,64]
VR	Immersive relaxation environments (eg, TRIPP)	Anxiety reduction, stress management, mindfulness, and relaxation	[10,27,28,50]
VR	Structured therapeutic platforms (eg, CBT^c^ modules)	Obsessive-compulsive disorder, schizophrenia, addiction treatment, and targeted therapeutic goals	[7,14,62]
Metaverse	Personalized digital avatars and twins	Depression, social anxiety, and personalized therapy through anonymity and emotional engagement	[7,47,50]
Metaverse	Immersive environments	Integrative platforms for meditation, peer-to-peer, and group therapy	[10,21]
Metaverse	Adaptive schema mode for immersive social interaction	Personality disorder treatment through immersive experiences	[21,43]
AR^d^	AR-based tools for neurofeedback and cognitive rehabilitation	Attention-deficit/hyperactivity disorder, dementia, and brain injury recovery	[8,41]
AR	Interactive AR environments	Social skills training for autism and social anxiety	[3,35]
AI-XR chatbots	Chatbots (eg, Woebot, Wysa, and Joyable) in XR	Delivering CBT and emotional support in interactive XR settings	[10,49]
XR	Biosensing technologies (eye tracking, photoplethysmography, electrodermal activity, and wearables)	Real-time stress management, trauma recovery, and neurofeedback therapy	[27,42]
XR	Dance movement therapy a	Nonpharmacological interventions for anxiety and depression	[60]
XR	Multimodular tools (eg, Multimodal Motion-Assisted Memory Desensitization and Reconsolidation)	PTSD and trauma recovery leveraging multisensory inputs for memory processing	[10]
MR^e^	Gamified XR and AI-driven therapy	Telerehabilitation and cognitive training	[26,41]
MR	Digital technologies for self-regulation	Meditation, mindfulness, addiction recovery, and emotional regulation; chronic pain and acute pain episodes	[55]
MR	Embodied AI-driven CBT	Therapeutic interventions leveraging embodied AI	[2,14,16]
MR	Immersive health care diagnostics with AI and XR	Diagnostics and therapeutic advancements in immersive environments	[44]

^a^VR: virtual reality.

^b^PTSD: posttraumatic stress disorder.

^c^CBT: cognitive behavioral therapy.

^d^AR: augmented reality.

^e^MR: mixed reality.

While AI-XR technologies hold immense promise for mental health care, several challenges must be addressed to fully realize their potential. Issues such as cybersickness, the need for therapist training, and the ethical handling of sensitive biofeedback data remain critical barriers. Future research should prioritize investigating long-term therapeutic outcomes and optimizing user experience to enhance the efficacy and accessibility of these interventions.

In summary, AI-XR technologies represent a transformative approach to mental health therapy, with diverse applications including exposure therapy, mindfulness, and personalized interventions. However, addressing existing challenges and advancing research are essential to ensure their effective integration into clinical practice and to maximize their therapeutic impact.

### RQ2: Challenges of Adopting AI-XR in Mental Health

The integration of AI-XR technologies into psychotherapy and mental health care presents major challenges that must be addressed to ensure their responsible and effective use. These challenges span ethical, psychological, relational, and cognitive domains, as outlined in the subsequent sections.

#### Ethical Challenges in AI-XR Psychotherapy

AI-XR systems in psychotherapy raise critical ethical concerns, particularly regarding data privacy and identity verification. The collection of sensitive health data in immersive environments creates complex data flows that challenge regulatory oversight, increasing risks of data breaches and unauthorized access [22,40,48]. The use of avatars and AI-driven systems further complicates user identity verification, potentially enabling impersonation and undermining therapeutic integrity [20,48]. Secure authentication protocols, such as multifactor authentication and biometric verification, are essential to safeguard patient privacy and build trust in these platforms [68]. Algorithmic bias is another pressing ethical issue, as AI systems trained on nonrepresentative datasets can produce biased treatment outcomes, disproportionately affecting marginalized groups and exacerbating health inequities [7]. In addition, ensuring informed consent is more complex in AI-XR therapies, as patients must understand both the technology and its potential biases. Clinicians must prioritize transparency during the consent process to enable informed decision-making [58]. The digital divide further complicates AI-XR adoption, as individuals in low-income or rural areas often lack access to necessary technologies [37]. In addition, AI-XR systems frequently fail to address the diverse needs of populations with disabilities or older adult users, highlighting the need for inclusive design to prevent exacerbating existing inequalities [66]. However, many inclusive features remain underdeveloped, leaving certain populations underserved [37,58].

#### Risk of Overload, Emotional Exposure, and Psychological Vulnerability

One emerging concern in AI-XR mental health applications is digital therapeutic dependency, where patients become overly reliant on AI-driven interventions, potentially reducing their engagement in real-world therapeutic processes [27]. This risk is heightened when AI-XR tools offer high levels of personalization, leading to excessive reliance on virtual environments rather than real-life coping strategies. Overuse of AI-XR in mental health care can lead to emotional overload and cognitive strain, particularly for individuals with anxiety or phobias. Excessive reliance on virtual environments for emotional regulation may result in isolation and negative psychological effects [21,31,58]. Effective therapy protocols must include boundaries and recovery periods to prevent overstimulation and ensure patients integrate virtual experiences into real-world contexts. The potential for maladaptive escapism in AI-XR environments poses risks for users with mental health issues, as excessive personalization could reinforce unhealthy coping mechanisms. In addition, avatars and digital personas may exacerbate body image issues or identity dissociation, leading to psychological harm [46,69,70]. Careful management of personalization is essential to mitigate these risks while maintaining therapeutic benefits [54]. Concerns remain around offensive behaviors and virtual assaults [31,84]. Furthermore, cybersickness, caused by sensory incongruences in XR environments, can lead to nausea, dizziness, and discomfort, underscoring the need for advancements in XR design to minimize these effects [2,71].

#### Issues in Preserving Patient-Therapist Relationships

While AI-driven therapies can improve accessibility, they risk undermining the human connection essential to effective psychotherapy. Automation may reduce the quality of care by replacing empathetic, nuanced human interactions with automated systems. Although AI tools such as chatbots offer consistent support, they cannot replicate the emotional depth of human therapists [66,72,73]. Balancing technological advancements with the preservation of therapeutic relationships is crucial to ensure patients’ well-being [33,65,74]. Ultimately, while technology can enhance accessibility and offer valuable support, maintaining the genuine human connection at the heart of therapy remains challenging. As AI and automation become more integrated into mental health care, it is crucial to focus on preserving the emotional and relational aspects of psychotherapy to ensure patients’ well-being is not compromised.

#### Clinician Perceptions and Resistance

Mental health professionals play a critical role in the adoption of AI-XR interventions, yet their acceptance is influenced by factors such as perceived efficacy, ethical concerns, and the potential disruption of traditional therapeutic models. Some clinicians express skepticism about AI-driven systems’ ability to provide meaningful therapeutic interactions, fearing that automation may undermine the human elements essential to psychotherapy [34,75]. In addition, concerns about liability and professional accountability in AI-assisted care contribute to hesitancy among practitioners [62]. Addressing these concerns through clinician education, transparent AI decision-making processes, and ethical guidelines is essential for fostering trust in AI-XR applications.

#### Patient Trust and Adoption Barriers

Beyond clinician resistance, patient mistrust presents another significant challenge. Many individuals express skepticism toward AI-driven therapy due to concerns about data privacy, the impersonal nature of digital interventions, and the perceived lack of emotional depth in AI-generated responses [85]. For vulnerable populations, such as those with severe mental health conditions, the idea of engaging with AI-XR systems instead of a human therapist may raise concerns about misdiagnosis, lack of empathy, or inappropriate treatment recommendations [53,63,86]. Ensuring transparency in AI decision-making and offering hybrid care models—where AI-XR complements, rather than replaces, human therapists—can help mitigate these concerns [75].

#### Cognitive and Epistemic Limitations of LLMs and AI-XR Technologies

LLMs such as GPT-based systems, despite their ability to simulate empathy, lack the cognitive and epistemic depth of human therapists. Their responses are based on statistical patterns rather than a genuine understanding, which may mislead patients or provide inadequate emotional support [22,48,74]. Researchers recommend using LLMs as support tools rather than replacements for human therapists, emphasizing the need for regulatory frameworks to ensure their safe and effective use in mental health care [7,57,59].

#### The Need for Cross-Disciplinary Collaboration

The adoption of AI-XR in mental health requires an interdisciplinary approach, where health care professionals, technologists, and policy makers work together to design, regulate, and implement effective solutions [55,78]. Engineers must develop systems that are clinically validated and ethically sound, while policy makers must establish regulatory frameworks that ensure data security, algorithmic fairness, and equitable access. Ethical oversight, particularly in addressing algorithmic bias and ensuring inclusivity, is essential to prevent AI-XR technologies from exacerbating health disparities [30]. Collaborative efforts that integrate clinical expertise with technological advancements will be key to maximizing the benefits of AI-XR while addressing its limitations.

From the aforementioned exploration, we understand that the ethical and social implications of AI-XR technologies in mental health treatment are multifaceted, encompassing issues of data privacy, algorithmic bias, digital inequality, informed consent, psychological impact, inclusivity, and regulatory oversight, as illustrated in Table 6.

**Table 6:** Research question 2: ethical and social issues in artificial intelligence–driven extended reality (AI-XR) technologies for mental health applications.

Mental health domain	Ethical and social issues	References
General mental health	Ethical concerns with user data, inclusivity in access, and digital inequality	[38,55]
Psychiatric care	Algorithmic bias leading to inequitable care and challenges with informed consent in immersive environments	[7,58]
Therapy	Challenges in preserving patient-therapist relationships, lack of empathy in AI therapy, and risks of screen-mediated therapy feeling impersonal	[53,72,73]
Large language models in therapy	Issues with bias, misinformation, overreliance on AI-generated insights, and inability to address nuanced emotional or psychological needs	[7,40,59,65]
Immersive experiences	Psychological impacts, such as maladaptive escapism, dependency, virtual traumas, dual representations, cybersickness	[46,54,69,70]
Digital well-being	Privacy concerns, digital inequalities, risks of harmful content, and overreliance on virtual therapies	[27]
Virtual medical consultations	Data security concerns and challenges with identity verification in virtual environments	[52]
Public health and remote therapy	Data privacy risks, potential digital addiction, and overreliance on AI-driven virtual therapy platforms	[41]
Surgical precision and remote care	Risks of digital divides, data security, privacy concerns, and inequality in accessing advanced technologies	[39]

Ethical governance frameworks prioritizing patient privacy, transparency, and inclusivity are necessary to guide the deployment of these technologies in a way that fosters trust and benefits all patients equitably [40]. We will explore these in the following subsections.

### RQ3: Regulatory Frameworks for AI-XR in Mental Health Treatment

#### Overview

As AI-XR technologies become integrated into mental health care, ensuring compliance with established regulatory frameworks is critical for safeguarding patient rights and maintaining ethical standards. Existing regulations such as the General Data Protection Regulation (GDPR) and the Health Insurance Portability and Accountability Act (HIPAA) provide guidelines for handling sensitive health data, enforcing privacy protections, and ensuring informed consent in digital health care settings. GDPR mandates explicit user consent, data minimization, and the right to erasure, while HIPAA focuses on safeguarding patient health information from unauthorized access and misuse [79,80]. However, the immersive nature of XR environments introduces new challenges, requiring updated policies that address real-time data collection, biometric tracking, and AI-driven decision-making. Ensuring AI-XR technologies uphold human dignity and clinical oversight is crucial in mental health care [17]. Regulatory frameworks must address key ethical concerns, including equitable access, data privacy, and algorithmic bias, while safeguarding care quality and maintaining professional accountability [87].

#### Integrating Fairness and Transparency

Developing trustworthy AI-XR systems requires addressing both technical and ethical complexities. Mitigating algorithmic bias necessitates diverse and inclusive data collection to prevent disproportionate outcomes [29,75]. Regular audits can identify biases, recalibrate decision-making models, and enhance accountability. Explainable AI (XAI) principles are essential for clinician and patient trust, ensuring that AI-driven decisions remain interpretable and transparent [34,63]. Simplified consent mechanisms, incorporating visual aids and plain language, improve patient comprehension [2,57,76]. Continuous reconsent protocols uphold patient autonomy in long-term AI-XR treatments, addressing ethical concerns around ongoing informed consent. AI-driven exposure therapies offer personalized interventions that dynamically adjust to patient responses, optimizing therapeutic outcomes [2,57]. Transparency and interpretability in AI models, particularly in conversational agents, are critical to fostering trust among patients and clinicians [57]. Intuitive interface designs and user guides help reduce technological barriers for nonexpert users. Empathy remains a challenge in AI-XR psychotherapy, as sentiment analysis tools require continuous refinement to improve contextual accuracy and sensitivity [29,45,63]. Lexicon-based and rule-based systems must adapt to evolving linguistic patterns to enhance reliability [85]. Data quality and representation are fundamental, as pretrained models must be rigorously adapted to mental health contexts to prevent biases and ethical violations [51,88].

#### Inclusive Design and Accessibility in Metaverse-Based Psychotherapy

Standardizing clinician accreditation for immersive therapies and enforcing stringent data security measures will foster public trust. Real-time auditing, dynamic informed consent processes, and interdisciplinary collaborations are essential for creating robust governance models for AI-XR in mental health care [78]. Inclusive design is vital for enhancing accessibility in AI-XR mental health technologies. Customizable features accommodate individuals with disabilities and diverse cultural backgrounds, improving engagement and therapeutic outcomes [49]. Automated therapy delivery systems can lower costs and expand access, particularly in underserved regions. Adaptive AI personalizes interventions based on patient needs, improving treatment effectiveness. AI-powered medical technology provides data-driven treatment recommendations for mental health conditions such as depression and PTSD [37,38]. However, rigorous regulatory oversight is necessary to ensure transparency, mitigate bias, and protect patient confidentiality. The digital divide continues to hinder equitable AI-XR deployment, necessitating targeted interventions such as subsidized infrastructure and culturally adaptive content to ensure inclusivity [80].

#### Regulatory Oversight, Mental Health Ethics, and AI-XR Governance

Beyond general data protection laws, mental health ethics guidelines, such as those from the American Psychological Association and the World Health Organization, provide ethical principles for AI-assisted therapy. These frameworks emphasize patient autonomy, transparency in AI decision-making, and clinician oversight in AI-driven interventions [50]. Ensuring that AI-XR aligns with these principles is essential to maintaining therapeutic integrity and preventing unintended harm. Regulatory frameworks must extend beyond data privacy concerns to enforce ethical standards and clinical oversight [48]. Certification programs and ethical review boards can validate AI-XR platforms for mental health care, ensuring accountability for developers and equitable access to effective treatments [17,77]. Embodied AI therapies, including avatars and virtual counselors, require continuous monitoring to prevent overreliance and emotional dependency [46,69,89]. Combining AI-driven interventions with human therapist supervision ensures balanced therapeutic engagement. Session caps and scheduled breaks are implemented to encourage real-world application of therapeutic skills. Patients are informed about AI’s role as a supplement, not a replacement, for human therapy. Establishing guidelines to prevent excessive AI dependency, similar to existing mental health treatment protocols. Long-term studies are necessary to assess their effects and ensure a balanced integration of virtual and real-world interactions [33]. Age-based content filters and disclaimers should be implemented to enhance safety and transparency in AI-driven psychotherapy tools [77]. Collaboration with safety experts is crucial for developing adaptive regulatory policies. Drawing from the World Health Organization Global Strategy on Digital Health (2020-2025), regulatory frameworks should incorporate ethical, legal, and operational safeguards [80].

#### Data Protection and Security Concerns

AI-XR technologies process sensitive mental health data, raising substantial concerns regarding confidentiality and security [28,62]. Secure data management is critical, with blockchain and distributed ledger technology offering tamper-proof records to safeguard patient privacy [34]. These technologies enhance transparency and accountability while minimizing the risks of data breaches [79]. Table 3 outlines the key areas of mental health treatment that require regulatory oversight, highlighting the need for comprehensive strategies to protect patient welfare.

The legal and regulatory landscape for digital health care technologies remains fragmented, particularly those used in immersive psychotherapy (eg, the metaverse, AR, VR, and XR). As with telemedicine, these technologies face challenges related to cross-border governance, patient confidentiality, liability, and compliance with data protection laws. The European Union’s (EU) frameworks, such as the GDPR and the Cross Border Healthcare Directive, aim to standardize digital health services but often fall short in addressing the unique nuances of mental health care [80]. This lack of harmonized policies creates uncertainty for practitioners and erodes patient trust, potentially hindering the adoption of these innovative approaches. National-level variations in regulation, funding, and legal interpretations further exacerbate these issues, leaving clinicians with insufficient guidance for safe and effective practice [90].

#### Training Mental Health Professionals

In addition, the AI-XR platforms play a transformative role in training psychiatrists, psychologists, and therapists. Immersive environments such as PATIENT allow for interactive and practical learning experiences, enabling clinicians to practice diagnosing and treating patients in simulated scenarios [51]. These virtual tools can reduce training costs and increase accessibility, though equitable access must be ensured to prevent knowledge disparities [40].

The metaverse can simulate various psychological conditions, enabling clinicians to practice diagnosing and treating patients in a safe, controlled environment. Ford et al [7] emphasized the importance of equitable access to these virtual educational tools to prevent a knowledge gap between those with and without access to these advanced technologies [91]. By incorporating metaverse-based training, mental health professionals can gain hands-on experience treating patients through AI-XR systems, preparing them for real-world applications [35].

Finally, virtual group therapy platforms allow patients to participate in therapeutic sessions with others from diverse geographical locations, fostering a sense of community [14]. However, these platforms also present challenges, such as maintaining patient confidentiality in a group setting and ensuring that the technology does not unintentionally exclude individuals due to socioeconomic factors [92]. Regulatory frameworks must address these concerns by setting clear guidelines for data protection, identity verification, and accessibility. In addition, legal protections must ensure that patients maintain control over their data and participation in virtual group therapy sessions. Systemically addressing barriers through coordinated global efforts that align with ethical standards and human rights principles is needed. Strategies such as subsidizing infrastructure development, fostering digital literacy, and ensuring culturally relevant therapeutic content are crucial for creating a genuinely equitable digital mental health ecosystem. With comprehensive frameworks and intentional design, immersive technologies can transform mental health care, delivering innovative and compassionate solutions to diverse populations worldwide.

## Discussion

The findings from this review underscore the transformative potential of AI-XR technologies in mental health treatment while highlighting the ethical, social, and regulatory challenges that must be addressed to unlock their full benefits. Each RQ explored in this study contributes to a holistic understanding of the opportunities and risks associated with AI-XR applications.

### Issues in Expanding Therapeutic Potential Through AI-XR Technologies

AI-XR technologies show promise in revolutionizing mental health treatment by offering immersive, personalized environments that enhance flexibility and adaptability, surpassing traditional methods. Studies indicate that XR tools such as VR and AR, combined with LLMs, create real-time, responsive therapeutic settings [16,93]. However, despite promising early results, studies such as those by Kurata et al [26] and Akhtar [18] highlight a lack of long-term empirical data and real-world validation for XR-based interventions.

While preliminary research is promising, the scalability and reproducibility of AI-XR solutions remain uncertain. Moodley et al [15] cited technical challenges, scalability issues, and regulatory hurdles that impede broader adoption. For example, despite offering many advantages, VR interventions are still constrained by high hardware demands, financial limitations, and usability challenges [47]. Akhtar [18] emphasized the scarcity of real-world data, particularly for long-term treatments, and ethical concerns surrounding the use of AI with vulnerable populations.

Moreover, many studies have limitations such as small sample sizes, short intervention durations, and overlooked side effects such as cybersickness. Mitsea et al [55] recommend using standardized tools such as the Simulator Sickness Questionnaire to assess these adverse effects. In addition, existing literature shows limited replication of findings, with inadequate exploration of emotional regulation and stress management [63,86]. Studies often focus on executive functions and socialization, neglecting the potential risks of excessive exposure or emotionally taxing interactions such as cyberbullying [54]. This highlights the need to safeguard patients and establish ethical virtual therapy practices.

An overreliance on technology in mental health care presents additional challenges, as individual needs require more adaptive, nuanced treatments. Automated VR cognitive therapy models, such as those tested by Freeman et al [49], offer consistent therapy delivery independent of therapist variability. The THRIVE trial and initiatives such as gameChange [94] illustrate the scalability and real-world potential of automated VR interventions. However, despite their scalability, these models must undergo rigorous validation across diverse populations and settings to ensure their effectiveness.

### Challenges in Remote and Chatbot-Based Therapies

While AI-driven psychotherapy chatbots show promise, they currently face limitations in adapting to immersive and multimodal virtual environments. An important challenge is the overreliance on text-based communication, which restricts user engagement in rich, interactive settings. Existing systems exhibit low compliance rates due to their static nature, which does not align with users’ expectations for dynamic communication [65]. To improve engagement and compliance, future chatbots must evolve beyond static, text-based models by integrating voice, gesture, and emotional recognition technologies. These enhancements would enable chatbots to interpret users’ intentions more accurately and improve engagement, satisfaction, and compliance. Furthermore, extending conversational depth and providing highly personalized therapy plans can significantly enhance long-term effectiveness and treatment outcomes [51].

Another pressing issue is the lack of robust danger detection mechanisms. In virtual environments, where users may exhibit heightened emotional states due to anonymity or detachment, chatbots must be equipped to recognize signs of self-harm or suicidal ideation [45]. Emotion recognition algorithms, capable of analyzing text, voice, and other multimodal inputs, can detect risky behaviors and initiate timely interventions. Misunderstandings caused by ambiguous inputs or insufficient contextual awareness further compound the challenge. By integrating contextual understanding from multimodal data—such as body language or avatar expressions—chatbots can deliver more accurate and empathetic responses, improving therapeutic outcomes.

In addition, remote and immersive technologies offer transformative potential in extending health care accessibility, particularly in underserved or crisis-affected areas. Innovations such as remote surgeries, telemedicine, and robot-assisted therapies have proven effective in addressing medical needs in rural regions with limited resources [66,89]. Virtual psychotherapy platforms, enhanced by AI-XR technologies, provide scalable solutions for trauma and mental health treatment in conflict-affected regions such as Gaza, Lebanon, Yemen, Sudan, and Ukraine [95]. These solutions can be vital for humanitarian organizations such as UNICEF, enabling them to deliver mental health support in remote, resource-constrained environments. However, for these technologies to reach their full potential, inclusivity and accessibility must be prioritized. Systemic barriers such as digital inequality, language limitations, and cultural sensitivity need to be addressed to ensure equitable access for all individuals, regardless of socioeconomic status or geography [96].

### Addressing Ethical and Social Challenges

The integration of AI and XR technologies into mental health care presents various ethical concerns, particularly around data privacy, autonomy, and long-term psychological effects [33]. Extended use of virtual environments, such as the metaverse, may impact self-perception, identity, and real-world relationships, particularly among vulnerable populations, such as adolescents and individuals with anxiety or psychosis [11,39]. These risks necessitate further research into the psychological impact of prolonged virtual immersion. Cultivating supportive virtual spaces that foster positive interactions, extending beyond the virtual realm, is essential to mitigate potential negative outcomes [54]. The integration of AI-driven avatars in therapy introduces concerns about patients becoming overly dependent on virtual therapists, which could diminish the critical role of human connection in the healing process [58]. To preserve the humanistic essence of psychotherapy, it is essential to establish clear boundaries that limit the scope of virtual interactions. For instance, virtual therapists could be positioned as supplemental tools to human therapists rather than replacements, ensuring that they assist with specific tasks such as skill-building or preliminary assessments while reinforcing the primacy of human-led sessions. These boundaries help minimize overreliance on AI-driven avatars by maintaining a balanced therapeutic approach that prioritizes genuine human connection.

In addition, social equity requires addressing the digital divide to ensure marginalized communities can access AI-XR–driven mental health care. Bridging gaps related to internet access, cultural content relevance, and device design is crucial for equitable participation across diverse populations [7,37]. Decentralized platforms in the metaverse could empower these communities, allowing for more culturally sensitive approaches to mental health care. Regulatory frameworks for AI-XR technologies in mental health care are still in development and must evolve to address emerging risks. Establishing standards for data privacy, transparency, and fairness will be key to fostering trust in these systems [29,90]. To maintain human-centered care, AI-XR technologies should be positioned as adjunct tools that support—rather than replace—clinicians, ensuring continuity of trust and emotional safety [93]. Furthermore, establishing trusted digital identities and safeguards against misuse will help preserve patient safety in virtual environments.

### Clinical, Technological, and Policy Recommendations

The findings from this review suggest several actionable insights for clinical practice, system design, and regulatory development.

#### Clinical Implications

AI-XR technologies hold transformative potential in clinical mental health care, particularly through telepsychiatry, virtual CBT, and immersive exposure interventions [80]. Their integration can help address professional shortages, reduce geographical barriers, and enhance continuity of care for patients in remote or underserved areas. However, clinical protocols must be adapted to the unique challenges of virtual care—such as managing cybersickness, assessing therapeutic alliance in immersive contexts, and determining the need for human intervention [47,55].

Training health care professionals in the effective use of these technologies and establishing clear clinical guidelines will be essential. Human oversight remains critical in immersive environments to mitigate risks and uphold the principle of nonmaleficence [86]. Developers must ensure that the benefits of immersive technologies, including VR and AI-driven chatbots, clearly outweigh potential risks—particularly in sensitive contexts such as treatment for depressive disorders [97]. Ultimately, rigorous testing, continuous updates, and human supervision are vital to ensuring that metaverse-based psychotherapy systems prioritize safety, equity, and therapeutic effectiveness [90].

#### Technological Considerations

From a system design perspective, AI-XR platforms must prioritize user-centered development—ensuring solutions are intuitive, accessible, and culturally sensitive. Addressing algorithmic bias is vital to avoid reinforcing existing health disparities. Developers should embed multimodal capabilities—such as voice, motion tracking, and emotional recognition—into AI chatbots and XR interfaces to enhance user engagement and therapeutic responsiveness.

Fostering metaverse literacy through targeted training can support ethical and informed use of these technologies. Patient involvement in cocreating their virtual therapeutic environments—by adjusting sensory elements, for example—can boost their sense of control, motivation, and self-efficacy. This active participation promotes deeper engagement and improved outcomes by empowering patients as partners in their healing journey.

#### Policy Recommendations

The ethical deployment of AI-XR in mental health care requires robust governance frameworks. Data protection policies must safeguard privacy and transparency, particularly for vulnerable populations. Regulatory alignment with frameworks such as the EU AI Act can help establish safety standards and accountability mechanisms [78]. In addition, it is critical to define the legal boundaries of AI-augmented therapy—distinguishing clinical from nonclinical use—and to require real-time monitoring and auditability of AI decisions.

Investments in digital infrastructure, public education, and metaverse literacy initiatives can help bridge the digital divide and promote equitable access [30]. Transparent communication about AI limitations and active strategies to mitigate dependency risks will protect patient interests. Furthermore, standards for AI-XR content must ensure evidence-based practices, real-time moderation, and cultural appropriateness [30,52].

As AI-XR technologies evolve, regulatory frameworks must remain adaptive. Traditional models may not suffice in addressing the rapid pace of innovation, necessitating a dynamic, collaborative approach among technologists, clinicians, and policy makers [36]. Future regulatory systems may benefit from real-time policy adjustment tools to respond to emerging applications while safeguarding patient safety and care quality [30].

### Limitations

This scoping review has several limitations. First, our search strategy did not include the Web of Science database, which may have led to the omission of relevant studies indexed exclusively in this source. However, we selected PubMed, PsycINFO, IEEE Xplore, Scopus, and Google Scholar to ensure broad interdisciplinary coverage of health sciences, psychology, and technology literature. In addition, many journals indexed in the Web of Science database are also covered by the Scopus, PubMed, and PsycINFO databases, and Google Scholar provided additional breadth by capturing gray literature and nonindexed studies. Nonetheless, we acknowledge that Google Scholar’s lack of advanced filtering and transparency in indexing may affect the reproducibility and methodological rigor of our search results. While we believe this approach minimized the impact of excluding Web of Science, we acknowledge that our methodology is not exhaustive.

Second, the review’s study selection and data analysis relied on manual processes, with duplicate screening conducted using Microsoft Excel. Although a second reviewer sampled the data, most of the extraction was performed by the first author. The use of more advanced systematic review tools could have enhanced methodological rigor, improved efficiency, and reduced potential errors in data synthesis.

Third, our search strategy included studies addressing mental health broadly but did not systematically target disorder-specific interventions. As a result, this review may not fully capture the breadth of AI-XR applications tailored to specific conditions, such as depression, anxiety, bipolar disorder, or PTSD. Future research should adopt a more granular approach by assessing AI-XR interventions for distinct mental health disorders, allowing a deeper understanding of their disorder-specific effectiveness.

In addition, the lack of standardized clinical efficacy metrics in AI-XR research poses a challenge. The studies reviewed used diverse assessment methods, making cross-study comparisons difficult. Establishing uniform evaluation criteria for AI-XR applications would improve the ability to assess their therapeutic impact reliably.

Furthermore, longitudinal research on AI-XR interventions is scarce. Most studies focus on short-term effects, leaving the long-term psychological, behavioral, and neurological implications of these technologies largely unexplored. Future research should prioritize longitudinal studies to assess sustained therapeutic benefits, potential risks such as digital therapeutic dependency, cybersickness, and changes in patient outcomes over time.

Finally, while this review highlights ethical concerns associated with AI-XR, empirical studies investigating real-world ethical, societal, and clinical implications remain limited. The impact of AI-driven interventions on therapist-patient relationships, algorithmic biases, and unintended psychological consequences warrants further investigation. Despite these limitations, this review provides a comprehensive overview of AI-XR applications in mental health care, identifying key research gaps and offering actionable recommendations for future studies.

### Future Directions

To advance the field of AI-XR interventions in mental health care, future research should adopt more targeted and actionable approaches. While this review highlights the potential of AI-XR applications, several critical areas require further investigation. By addressing these key areas, future research can contribute to the responsible development and implementation of AI-XR interventions, ensuring their efficacy, accessibility, and ethical alignment in mental health care.

#### Comparative Trials of AI-XR Versus Traditional Telehealth Approaches

Future studies should conduct randomized controlled trials comparing AI-driven XR interventions with conventional telehealth and in-person psychotherapy [32,49]. These trials can assess the relative effectiveness, patient adherence, and clinical outcomes of AI-XR solutions in treating mental health conditions such as depression, anxiety, PTSD, and bipolar disorder.

#### Disorder-Specific AI-XR Interventions

While AI-XR interventions have demonstrated promise across various mental health conditions, future research should delve deeper into disorder-specific applications to refine therapeutic approaches. Studies should investigate how AI-XR technologies can be tailored to distinct conditions such as schizophrenia, OCD, borderline personality disorder, eating disorders, and substance use disorders, which are currently underexplored in AI-XR literature. Comparative studies should assess the effectiveness of AI-XR interventions within each disorder category, ensuring that XR-based treatments align with disorder-specific cognitive, behavioral, and emotional characteristics.

#### Human-AI Hybrid Therapy Models

Research should explore the development and evaluation of hybrid models that integrate AI-driven XR tools with human therapists. This includes investigating how AI can augment, rather than replace, human expertise in psychotherapy by providing real-time insights, personalized treatment recommendations, and automated therapeutic interventions [75]. Key areas of focus should include patient acceptance, therapist-AI collaboration, and ethical considerations.

#### Scalability and Accessibility in Low-Resource Settings

The feasibility and effectiveness of AI-XR interventions in underserved and low-resource environments remain largely unexamined. Future studies should evaluate the adaptability of these technologies in settings with limited mental health infrastructure, internet connectivity, and digital literacy. Research should also examine cost-effectiveness, cultural considerations, and potential strategies to bridge disparities in access to AI-powered mental health care.

#### Longitudinal Studies on Digital Therapeutic Dependency

While AI-XR technologies hold promise for enhancing mental health care, prolonged exposure may contribute to digital therapeutic dependency, cybersickness, and maladaptive escapism where patients become overly reliant on virtual interventions [46,55,69]. Future research should investigate the psychological and behavioral implications of sustained AI-XR use, exploring strategies to mitigate dependency risks while maintaining therapeutic efficacy.

#### Regulatory and Ethical Frameworks

The integration of AI and XR into mental health care necessitates robust regulatory guidelines to ensure patient safety, data privacy, and ethical AI use. Future studies should analyze how existing regulations (eg, GDPR, HIPAA, and EU Medical Device Regulation [EU-MDR 2017/745]) apply to AI-XR applications and propose frameworks to address emerging ethical concerns, such as algorithmic bias, informed consent in virtual environments, and clinician accountability [41,79,80].

#### Personalization and Adaptive AI in Mental Health Care

Future work should also explore how adaptive AI models can tailor XR interventions to individual patient needs, dynamically adjusting content based on real-time physiological and behavioral data [54]. This includes investigating machine-driven personalization strategies that enhance user engagement, optimize treatment efficacy, and prevent adverse effects.

### Conclusions

This review is the first to comprehensively evaluate AI and XR applications in metaverse-driven mental health care, focusing on their therapeutic potential, ethical concerns, and societal impacts. Following the PRISMA-ScR guidelines, we analyzed 48 peer-reviewed studies published after 2014, selecting those that addressed clinical applications, ethical issues, and social implications of AI-XR, while excluding nonresearch articles and studies lacking relevance or accessibility.

Organized around 3 core RQs—therapeutic potential (RQ1), ethical challenges (RQ2), and societal implications (RQ3)—this synthesis highlights the transformative potential of AI-XR in mental health treatment and the governance frameworks needed for responsible deployment. For RQ1, our findings suggest that AI-XR applications such as VR therapy and AI-driven diagnostic tools enhance accessibility and patient engagement, particularly in resource-limited settings. However, large-scale, longitudinal studies are needed to validate their effectiveness across diverse mental health conditions and populations [98].

Regarding RQ2, ethical concerns such as privacy risks, data security, and algorithmic bias emerged as key challenges. The handling of sensitive biosignal data raises questions of data ownership and misuse, while bias in AI models could result in inequitable treatment for marginalized groups. These findings underscore the need for robust governance frameworks that prioritize transparency, informed consent, and privacy protections tailored to immersive mental health solutions [99]. For RQ3, we identified significant societal implications, including changes in patient-therapist dynamics, risks of digital exclusion, and psychological dependency on virtual environments. These insights highlight the necessity of inclusive regulatory policies to mitigate the long-term social and relational risks posed by AI-XR therapy.

### Call to Action: Bridging Innovation and Responsibility

To ensure ethical, effective, and equitable adoption of AI-XR in mental health care, targeted actions are needed from key stakeholders:

- Policy makers should establish AI-XR–specific regulations, aligning with existing frameworks (eg, GDPR, HIPAA, and MDR). Ensure data security, bias mitigation, and patient safety while supporting standardized ethical guidelines for AI in mental health.
- Cinicians and mental health professionals should embrace AI-XR tools as adjunctive rather than replacement therapies. Push for clinical validation, patient safety protocols, and AI literacy training in medical and psychological practice.
- Developers and researchers should design transparent, bias-aware AI models while prioritizing privacy, security, and ethical AI governance. Conduct comparative trials between AI-XR and traditional mental health interventions.

By collaborating across disciplines, we can harness the potential of AI-XR while ensuring its safe, ethical, and inclusive integration into global mental health care systems.

## Appendix

Multimedia Appendix 1 The PRISMA-ScR checklist.

Multimedia Appendix 2 Search strategy and search queries for all databases, along with a complete overview of the article screening process across all stages.

Multimedia Appendix 3 Data extraction form, including extracted characteristics, methodology, technology, and other attributes.

## Abbreviations

AI: artificial intelligence
AI-XR: artificial intelligence–driven extended reality
AR: augmented reality
CBT: cognitive behavioral therapy
GDPR: General Data Protection Regulation
HIPAA: Health Insurance Portability and Accountability Act
LLM: large language model
MR: mixed reality
OCD: obsessive-compulsive disorder
PRISMA-ScR: Preferred Reporting Items for Systematic Reviews and Meta-Analyses extension for Scoping Reviews
PTSD: posttraumatic stress disorder
RQ: research question
VR: virtual reality
VRET: virtual reality exposure therapy
XR: extended reality
