# Supplementary material for: Exploring the Application of AI and Extended Reality Technologies in Metaverse-Driven Mental Health Solutions: Scoping Review
Source: J Med Internet Res. 2025 Aug 19;27:e72400. doi: 10.2196/72400 (PMC12405795; doi:10.2196/72400)
Supplement: Multimedia Appendix 2 [file jmir_v27i1e72400_app2.docx]

| Monday, May 30, 2022 6:45:26 AM Monday, May 30, 2022 6:39:40 AM |
| --- |

**Appendix 2: Search strategy**

| **Database** | **Search Query** |
| --- | --- |
| **Google Scholar** | ("Artificial Intelligence" OR AI OR "AI-Driven" OR "Machine Learning") AND ("Extended Reality" OR XR OR "Virtual Reality" OR VR OR "Augmented Reality" OR AR OR Metaverse) AND ("Mental Health" OR Psychotherapy OR "Psychological Disorders") AND ("AI in Therapy" OR "Psychotherapeutic Outcomes" OR "Immersive Therapy" OR "Therapeutic Effectiveness") AND ("Ethical Dilemmas" OR "Privacy in AI" OR "AI Ethics" OR "Patient Safety" OR "Data Governance" OR "AI Decision-Making") AND ("Patient-Therapist Relationships" OR "Digital Exclusion" OR "Psychological Impact" OR "Virtual Environments in Therapy" OR "Outcomes" OR Benefit* OR advantage* OR effective* OR impact* OR improve* OR efficacy OR challenge* OR barrier* OR limitation* OR Privacy OR Safety OR Regulat* OR Societ* OR difficult* OR obstacle* OR ethic* OR "future direction*" OR trend* OR "research gap*") |
| **PsycINFO** | ("Artificial Intelligence" OR AI OR "Machine Learning" OR "AI-Driven" OR Metaverse) AND ("Mental Health" OR Psychotherapy OR "Psychological Disorders") AND (Effectiveness OR Outcomes OR "Ethical Dilemmas" OR "Privacy in AI" OR "AI Ethics" OR "Patient Safety" OR "Data Governance" OR benefit OR challenge OR issue) |
| **IEEE Xplore** | ("Extended Reality" OR "XR" OR "Virtual Reality" OR "VR" OR "Augmented Reality" OR "AR" OR "Metaverse") AND ("Mental Health" OR "Psychotherapy" OR "Psychological Disorders") AND ("Privacy" OR "Patient Safety" OR "Data Governance" OR "Ethics" OR "Ethical Challenges" OR "Societal Challenges" OR "Benefits" OR "Outcome") |
| **PubMed** | (("Virtual Reality"[Mesh] OR "Virtual Reality" OR "Augmented Reality" OR "Mixed Reality" OR "Extended Reality" OR XR OR "Immersive Therapy" OR "Digital Therapeutics") AND ("Psychotherapy"[Mesh] OR "Mental Health Treatment" OR "Cognitive Behavioral Therapy") AND ("Ethics" OR "Privacy" OR "Patient Safety" OR "Regulatory Frameworks" OR "Social Impact" OR "Data Governance") AND ("Therapeutic Benefits" OR "Outcome" OR "Accessibility" OR Benefits)) |
| **Scopus** | (TITLE-ABS-KEY ( "Artificial Intelligence" OR AI OR "AI-Driven" OR "Machine Learning" ) AND TITLE-ABS-KEY ( "Extended Reality" OR xr OR "Virtual Reality" OR vr OR "Augmented Reality" OR ar OR metaverse ) AND TITLE-ABS-KEY ( "Mental Health" OR psychotherapy OR "Mental Disorders" ) AND TITLE-ABS-KEY ( "Ethics" OR privacy OR "Data Governance" OR "Patient Safety" OR "Societal Norms" OR "Outcome" OR Benefits) ) |

| **DATABASE** | **ARTICLES WITH QUERY** | **YEAR 2014-2024 & ARTICLE TYPE** | **ENGLISH** | **OPEN ACCESS & DUPLICATES** |  |
| --- | --- | --- | --- | --- | --- |
| **Google Scholar** | 1110 | 749 | 634 | -  MANUAL SCREENING |  |
| **PsycINFO** | 21 | 17 | 17 | 13 |  |
| **IEEE Xplore** | 74 | 65 | 65 | 4 |  |
| **PubMed** | 35 | 30 | 26 | - |  |
| **Scopus** | 48 | 37 | 21 | 14 |  |
| **TOTAL** | 1288 | 898 (1288-390) | 763 (898-135) | 408 (763-355) | |
| **Screening** | 408 (-295) | | | | |
| **ELIGIBLE** | 113 (-9) | | | | |
| **FULL TEXT** | 104 (-63) = 41 (added 7) | | | | |
| **INCLUDED** | 48 | | | | |
