# Supplementary material for: Exploring the Application of AI and Extended Reality Technologies in Metaverse-Driven Mental Health Solutions: Scoping Review
Source: J Med Internet Res. 2025 Aug 19;27:e72400. doi: 10.2196/72400 (PMC12405795; doi:10.2196/72400)
Supplement: Multimedia Appendix 3 [file jmir_v27i1e72400_app3.docx]

| **Extracted data** | **Definition** |
| --- | --- |
| **Study Characteristics** |  |
| Author | The first author of the study (last name). |
| Year of publication | The year in which the study was published. |
| Type of publication | The venue where the study was published: peer-reviewed journal articles, conference proceedings, or dissertations. |
| **Study Methodology and Details** |  |
| Setting | Virtual environment, clinic, community, online platform, etc. |
| Key findings | Applications of AI, XR, VR, AR, Metaverse in mental health.  Benefits and impacts of these technologies.  Proposed frameworks or models for integration.  Guidelines for effective use |
| Study Design | • Observational, experimental, case study, or qualitative.  • Cross-sectional, longitudinal, or systematic review. |
| Relation to our study | Focus of the paper in relation to the research questions (e.g., Benefits, AI-driven therapy, ethical concerns, privacy issues) |
| **Technology characteristics** |  |
| Technology Used | • Artificial Intelligence (AI)  • Extended Reality (XR)  • Virtual Reality (VR)  • Mixed Reality (MR)  • Augmented Reality (AR)  • Metaverse |
| Names of the tools used for mental health treatments | List specific AI tools or platforms mentioned in the study, e.g., IBM Watson, AI-powered chatbots, virtual therapy platforms, etc. |
| AI methods (if applicable) | • Machine learning algorithms (e.g., supervised learning, reinforcement learning)  • Natural Language Processing (NLP), Large Language Models (LLMs)  • Deep learning (e.g., neural networks), GenAI |
| **Healthcare Domain** |  |
| Psychotherapy | Does the study focus on AI or XR applications within psychotherapy settings (e.g., virtual therapy, AI-driven cognitive behavioral therapy)? |
| Mental healthcare | How does the study address broader mental health concerns (e.g., treatment of anxiety, depression, PTSD using AI/XR/VR tools)? |
| Outcomes and Evaluation |  |
| Outcomes Measured | • Clinical outcomes (e.g., symptom reduction, mental health improvement).  • Patient satisfaction, engagement, adherence, and usability.  • Therapist feedback on AI or XR tools. |
| **Evaluation Metrics** | • Efficacy (e.g., pre-and post-intervention measures).  • Psychological impact (e.g., changes in anxiety, depression, PTSD symptoms). |
| Challenges and Limitations |  |
| **User Experience:** | Barriers in technology adoption (e.g., patient comfort, ease of use, accessibility). |
| **Ethical issues** | • Data storage practices (e.g., encrypted, anonymized).  • Handling of sensitive patient data (e.g., compliance with HIPAA, GDPR). |
